# Factors influencing decision-making by social care and health sector professionals in cases of elder financial abuse

**DOI:** 10.1007/s10433-013-0279-3

**Published:** 2013-04-27

**Authors:** Miranda L. Davies, Mary L. M. Gilhooly, Kenneth J. Gilhooly, Priscilla A. Harries, Deborah Cairns

**Affiliations:** School of Health Sciences and Social Care, Brunel Institute for Ageing Studies, Brunel University, Uxbridge, UB8 3PH UK

**Keywords:** Elder financial abuse, Decision-making, Bystander intervention, Safeguarding, Social care, Health care

## Abstract

This study aimed to identify the factors that have the greatest influence on UK social care and health sector professionals’ certainty that an older person is being financially abused, their likelihood of intervention, and the type of action most likely to be taken. A factorial survey approach, applying a fractional factorial design, was used. Health and social care professionals (*n* = 152) viewed a single sample of 50 elder financial abuse case vignettes; the vignettes contained seven pieces of information (factors). Following multiple regression analysis, incremental *F* tests were used to compare the impact of each factor on judgements. Factors that had a significant influence on judgements of certainty that financial abuse was occurring included the older person’s mental capacity and the nature of the financial problem suspected. Mental capacity accounted for more than twice the variance in likelihood of action than the type of financial problem. Participants from social care were more likely to act and chose more actions compared to health sector participants. The results are discussed in relation to a bystander intervention model. The impact of the older person’s mental capacity on decision-making suggests the need for training to ensure action is also taken in cases where older people have full mental capacity and are being abused. Training also needs to highlight the more subtle types of financial abuse, the types that appear not to lead to certainty or action.

## Introduction

Elder financial abuse, which may be defined as ‘theft, fraud, exploitation, pressure in connection with wills, property or inheritance or financial transactions, or the misuse or misappropriation of property, possessions or benefits’ (Department of Health 2000, Section 2.7, p. 9), is widely regarded as a major social problem and one which is likely to grow with the ageing of societies throughout Europe and beyond (World Health Organization 2011). Current prevalence figures, which range from 0.7 % (O’Keeffe et al. 2007) to 14.4 % (Cohen et al. 2007; De Donder et al. 2011a, b), are believed to represent the ‘tip of the iceberg’ (National Center on Elder Abuse 1998). How professionals who have contact with older people detect such abuse and decide what to do is largely unexplored. This study aimed to address this gap by examining the decision-making of social care and health sector professionals in the United Kingdom (UK) regarding certainty that an older person is being financially abused, likelihood of taking action and the actions they would most likely take.

In the UK professionals working in the social care sector receive extensive training concerning adult safeguarding and so would be expected to be actively involved in identifying and responding to cases of elder financial abuse as part of their professional responsibilities. The role of health professionals in relation to adult safeguarding is receiving increasing emphasis, but there is no requirement for health professionals to share information with other agencies such as social services regarding suspicions of elder abuse generally, let alone financial abuse. Importantly, in the UK there is no mandatory reporting of suspicions of elder financial abuse; indeed, there is no absolute requirement to report and act upon suspicions of any kind of elder abuse even by those working in adult safeguarding.

Even if there was a requirement to report suspicions of financial elder abuse, compared to other forms of elder abuse, financial abuse is thought to be particularly difficult for professionals to identify. Money management practices are not commonly addressed as part of social care practice (Wilson et al. 2009). For health care professionals such as general medical practitioners (GPs), active identification of elder financial abuse is also not seen as a central part of the job role (Lachs and Pillemer 1995).

### Decision-making and elder abuse

Despite the highlighted need in the UK for evidence-based guidelines to support elder abuse detection and prevention by social and health care professionals (Department of Health 2000), research exploring decision-making by professionals working in the health and social care sectors in the context of elder abuse is limited (Killick and Taylor 2009). There is a body of literature describing various forms of abuse and their prevalence (Cooper et al. 2008) and indicating risk factors for elder abuse (De Donder et al. 2011a, b; Choi et al. 1999; Dong and Simon 2010; Marmalejo and Penhale 2011), with attempts to develop screening tools (Cohen 2011; Yaffe et al. 2008), but little evidence as to how professionals weigh up the importance of different factors when deciding if abuse is taking place and when deciding whether or not to intervene.

This lack of decision-making research in the field of financial elder abuse suggested a need for a multiple phase research project to, first, establish the factors that lead social care and health professionals to detect elder financial abuse and, secondly, determine how this information influences decision-making surrounding preventative action.

### Project background

Professionals working in the social care, health and banking sectors were involved in an elder financial abuse research project consisting of three phases, of which this study constitutes Phase II. The project as a whole explored the utility of the ‘bystander intervention’ model to explain why elder financial abuse often goes unreported (Gilhooly et al. 2013). Although developed to explain why people fail to act in emergencies (Darley and Latané 1968; Latané and Darley 1968, 1970, 1976; Latané 1981; Latané and Nida 1981), the bystander intervention model has considerable potential to help us understand decision-making in relation to the detection and prevention of elder financial abuse. There are five stages to our modified ‘professional bystander intervention model’ (Gilhooly et al. 2013):

1. noticing relevant cues to financial abuse,
2. construing the situation as suspected financial abuse,
3. deciding the situation is a personal responsibility,
4. knowing how to deal with the situation and
5. deciding to intervene.

Using qualitative methods, Phase I of our project aimed to establish the range of case features (decision cues) that professionals use in identifying elder financial abuse. Phase I revealed a wide range of case features that lead professionals to conclude that financial abuse is occurring. Social care and health sector study participants were found to use similar cues, whereas those in banking reported different cues (Gilhooly et al. 2013; Davies et al. 2011). For example, the types of abuse detected (noticed) by participants in banking were quite different from health and social care participants. Mental and physical capacity was reported as an important case feature by health professionals, but rarely mentioned by banking participants. The ‘money controller’ was a key decision cue for study participants from banking, but was never mentioned by participants from health and social care. Interesting as these findings were [and further details can be found in Davies et al. (2011) and Gilhooly et al. (2013)], what the findings from Phase I could not tell us was how the case features were weighted when deciding if financial abuse was definitely taking place or when deciding whether or not to intervene.

Phase II, therefore, aimed to investigate, using quantitative methods, which of these financial abuse case features had the most influence on social care and health sector professionals’ judgements. Phase III examined the relationship between policy guidance and what happens in practice and will be reported in a separate publication.

## Aims

The following research questions were addressed:

1. Which case features explain the greatest variance in certainty that elder financial abuse is taking place?
2. Which case features explain the greatest variance in likelihood of taking action in cases of suspected elder financial abuse?
3. What is the relationship between certainty that abuse is occurring, likelihood of taking action and action taken?

## Methods

### Design

A factorial survey approach was chosen to investigate judgements in cases of elder financial abuse to represent the range of factors that may be present in a situation of suspected abuse and to model the relationship between factors that raise professionals’ suspicions and resulting decisions (Taylor 2006). This approach assesses a professional’s judgements of a series of elder financial abuse case vignettes, to measure how many pieces of information (factors) are used to reach a judgement, and how the importance of the different factors is weighted (Rossi and Nock 1982).

### Participants

The sample included 152 professionals from the social care and health sectors. Social care professionals (total *n* = 70) included registered managers (*n* = 20), social care management level professionals (*n* = 12), social workers (*n* = 12), safeguarding adults managers (*n* = 9), directors/managing directors (*n* = 7), care managers (*n* = 6) and social work team managers (*n* = 4). Health professionals (total *n* = 82) included occupational therapists (*n* = 33), general practitioners (*n* = 17), practice managers (*n* = 15), nurses (*n* = 10) and administration roles (*n* = 7). There were no significant differences between social care and health sector participants in terms of age (43.9 and 41.5 years), gender (75.7 and 79.3 % female), ethnicity (88.6 and 80.5 % white) or years in the profession (12.4 and 12.0 years).

In order to access social care and health sector professionals working in inner city, suburban and rural areas, respectively, participants were recruited in the UK from North West London, South West London, and the counties of Kent, Hampshire and Medway. These recruitment areas were consistent with the area targeted in Phase I of the research (Davies et al. 2011; Gilhooly et al. 2013).

The recruitment of health professionals was facilitated by the Primary Care Research Network (PCRN) which approached all GP surgeries across the multi-site area with details of the research. PCRNs help researchers conduct research in primary care settings by supporting both recruitment and the logistics of carrying out research involving NHS staff. In the UK GP surgeries are based in the community, and carry out consultations with patients to deal with health issues or refer to other services for further treatment. A high proportion of consultations involve older patients. This method of recruitment aimed to create a simple random sample, with all health professionals working across the multi-site area equally likely to participate. Occupational therapists across the UK were also approached via the College of Occupational Therapists Specialist Section for Older People.

Social care professionals were recruited from local authority social care departments. The UK is made up of a series of councils that manage services in their local areas, such as adult social care. Professionals were recruited from three councils across the recruitment area, including one county Council, and two London Borough Councils. Recruitment was limited to three councils because to go beyond three requires additional approval from the Association of Directors of Adult Social Services and the payment of a significant fee. The local authorities were chosen to reflect inner city, rural and suburban areas.

### Procedures

#### Elder financial abuse case vignettes

Seven factors were included in the case vignettes. Four vignette factors were derived from content analysis of professionals’ case experiences obtained in Phase I of the research and were: the nature of the ‘financial problem suspected’, the older persons’ ‘mental’ and ‘physical capacity’ and the ‘identifier of the abuse’. Consultation with social care and health sector professionals lead to the addition of the older persons’ ‘age’, ‘gender’ and ‘living circumstances’ to provide necessary contextual details to the cases. In the vignettes, the factors were presented in the following order: (1) age, (2) gender (3) identifier of the abuse, (4) the nature of the ‘financial problem suspected’, (5) physical capacity, (6) mental capacity, and (7) living circumstances. The factors and factor levels are presented in Table 1.

**Table 1:** Elder financial abuse vignette factors and levels

Factor	Levels
Age (years)	66/76/86/96
Gender	Male/female
Identifier of abuse	‘You notice’
	‘A family member tells you’
	‘They tell you themselves’
	‘Their friend tells you’
	‘Another professional tells you’
Financial problem suspected	*A relative concerned about loss of inheritance*: ‘a relative has objected to the house being sold to pay for her care needs because of the impact on inheritance’
	*Stealing from the home or person*: ‘no change had been given after the shopping was done for him/her’
	*Anomalies between finances and living conditions*: ‘there is very little money available for day-to-day necessities and the basics in the cupboards are the cheapest of the cheap’
	*Financial anomalies in accounts or bills*: ‘there has been a letter from the bank which shows an overdrawn account and other showing bills haven’t been paid’
	*Recent change to a person*’*s will*: ‘recently a change to his/her Will has been made, leaving all possessions to the cleaner’
	*Misuse of Power of Attorney authority*: ‘the lasting power of attorney is now managing his finances and money is missing from his current account’
	*Rogue traders*: ‘building work was recently paid for and hasn’t been carried out’
Physical capacity	No physical health problems/minor physical health problems/major physical health problems
Mental capacity	Fully mentally aware/at times slightly confused/extremely confused and forgetful
Living circumstances	In their own home/with family/in their own home with a care package^a^/in sheltered accommodation/in residential care/in a nursing home

^a^A ‘care package’ is services provided to someone based on a review of their situation to help them continue to live independently. For instance, help with cleaning or preparing food

Participants completed the task on line. The recruitment information provided participants with the web link to access the judgement task directly. To ensure that people did not complete the task more than once, email addresses and demographic details of participants who had completed the task were checked for uniqueness. No instances of duplicate participation were identified. Participants were given two examples of vignettes to judge before starting the full scenario set. Each participant viewed the same scenario set consisting of 50 financial abuse case vignettes presented in a randomised order.

#### Dependent variables

**Fig. 1:**
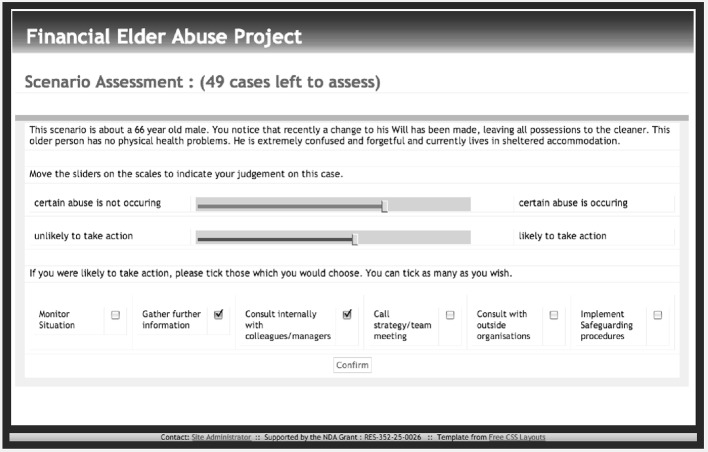
Example of an elder financial abuse case vignette

Participants were asked to make three separate judgements in response to each vignette. The first was to judge their certainty of financial abuse between two extremes from ‘certain abuse is not occurring’ to ‘certain abuse is occurring’, which represented a 0–100 scale. The second judgement was likelihood of taking action, ranging from ‘unlikely to take action’ to ‘likely to take action’, also on a 0–100 scale. Third, participants were asked to identify the actions they might take if faced with suspected elder financial abuse; participants were allowed to choose more than one action. An example case vignette and the web page set up can be seen in Fig. 1.

#### Fractional factorial design

A fractional factorial design was chosen to generate the sample of case vignettes. This approach reduces the cognitive load of the task for participants because it requires a minimal number of vignettes to be viewed, while enabling the separate impact of the factors to be established (Gunst and Mason 2009). All participants judged a single set of case vignettes, with the factor presentation devised to be both symmetrical and orthogonal (Dülmer 2007). Participants’ judgements were only included in the analysis if they responded to all the vignettes. Fractional-factorial sampling was carried out by author KG using SPSS, creating a case set of 50 elder financial abuse vignettes, in which implausible vignettes (such as a having a nursing home resident cheated by builders) were excluded. As a check on cue-independence, Lambda coefficients of association, which can vary between 0 (no association) and 1 (perfect association), were calculated between all the cues over the 50 vignettes. The average value was 0.08 with a non-significant maximum of 0.16 (between living circumstances and suspected type of abuse). These results indicated a satisfactory level of independence of the cues in the scenario set.

#### Analyses

Multiple regression analysis with blockwise entry was conducted to explore the extent to which judgements of certainty of elder financial abuse and likelihood of action could be predicted by vignette factor levels. Regression analysis was conducted at the overall level with the dependent variable representing the average scenario judgements across the sample.

Three of the elder financial abuse factors including ‘Identifier of abuse’, ‘Financial problem suspected’, and ‘Living circumstances’, were recoded into dummy variables whereby each factor level becomes an independent variable, coded as either ‘1’ or ‘0’, dependent on whether the factor level was present in the scenario. One factor level is chosen as a reference category and is excluded from the analysis, scored as ‘0’ for each dummy variable (Cooksey 1996). The reference category is the point of comparison for the relevant dummy variable categories (Hardy 1993). Because the interpretation of the regression analysis was tied to the specific reference categories selected, further analysis was needed to compare the overall impact of each factor. Incremental *F* tests were conducted, by running multiple regression analyses excluding each factor in turn. The *R* ^2^ for the model without each factor could then be subtracted from the *R* ^2^ for the model overall, to establish the squared semi-partial correlations (Cooksey 1996). The incremental *F* test (Hardy 1993) compares the *R* ^2^ for the reduced versus the full regression model to establish if there is a significant change in judgements as a result of the factor, accounting for the other factors present.

In order to compare the impact of the different factor levels on judgements, *t* tests of the regression coefficients were then conducted where the incremental *F* test identified that the factor had a significant influence on judgements (Hardy 1993). The Bonferroni correction was applied to determine an adjusted significance level to account for the fact that multiple *t* test comparisons were needed.

The number of times each action was selected by participants in response to each scenario was calculated. Frequency counts were obtained separately for social care and health professionals to compare if there was a difference between social care and health sector professionals in terms of their action choices. Independent sample *t* tests compared the percentage of times social care and health professionals selected each action across the 50 vignettes.

#### Ethical approval

Ethical approval for the research was obtained from Brunel University, and the South West NHS research ethics committee.

## Results

### Which case features explain the greatest variance in certainty that elder financial abuse is taking place?

Regression analysis revealed a significant impact of the financial problem suspected of ‘Rogue traders’, ‘Misuse of Lasting Power of Attorney authority’ and ‘Anomalies between finances and living conditions’. In addition, the older person’s ‘Mental capacity’ had a significant influence on professionals’ certainty of elder financial abuse (Table 2). If the remaining scenario factors are controlled, each increase in concern about mental capacity (e.g. from ‘Fully mentally aware’, to ‘At times slightly confused’) increases certainty of abuse by 7.7 %. The dummy variable results show the difference between each category and the reference group with the other scenario factors controlled. For example, where financial problems involved rogue traders, certainty of abuse was 10 % higher than for cases where a relative is concerned about loss of inheritance (the reference group) controlling for all the additional scenario factors.

**Table 2:** Regression model to identify the factors predicting social care and health professionals’ certainty of elder financial abuse

Factor (reference category)	Category	*B*	*SE B*	*P*
	Constant	42.34	8.13	0.00
Age		−0.05	0.08	0.54
Gender		0.75	1.72	0.67
Identifier of abuse *(*you)	Family	−2.08	2.44	0.40
	Professional	−0.79	2.21	0.72
	Subject	−3.10	2.38	0.21
	Friend	−2.74	2.15	0.21
Financial problem suspected	Change to the person’s will	3.21	2.96	0.29
(A relative concerned about loss of inheritance)	Stealing	0.63	2.84	0.82
	Anomalies in accounts or bills	−4.42	2.90	0.14
	Rogue traders	10.18	3.26	0.00***
	Misuse of POA authority	8.13	2.95	0.01**
	Anomalies between finances and living conditions	−6.43	2.86	0.03*

Consistent with the results in Table 2, incremental *F* test results (Table 3) showed that only the nature of the financial problem suspected, and the older person’s mental capacity explained a significant amount of the variance in certainty of abuse scores.

**Table 3:** *R* ^2^ change and *F* test results for each financial abuse factor predicting certainty of abuse and average likelihood of taking action

Factor	Certainty of abuse		Likelihood of action	
	*R* ^2^ change	*F*	*R* ^2^ change	*F*
Age	0.002	0.39	0.002	0.42
Gender	0.001	0.19	0.001	0.15
Identifier of abuse	0.013	0.67	0.013	0.63
Financial problem suspected	0.266	9.27***	0.153	4.96**
Physical capacity	0.012	2.46	0.016	3.10
Mental capacity	0.300	62.88***	0.381	74.01***
Living circumstances	0.008	0.34	0.011	0.44

** *P* < 0.01; *** *P* < 0.001

#### Comparing the influence of factors on social care and health professionals

Following the same process as for regression at the overall level, regression analysis was also conducted to identify any distinctions between factor weighting by profession. The results for regression analysis for the two groups separately were very similar to the overall regression findings, and the factors identified as significant by incremental *F* test analysis also followed the same pattern as the overall results (Table 4)

**Table 4:** *R* ^2^ change and *F* test results for each financial abuse factor predicting average certainty of abuse and likelihood of action for social care and health sector participants

Factor	Certainty of abuse				Likelihood of action			
	Social care		Health sector		Social care		Health sector	
	*R* ^2^ change	*F* test	*R* ^2^ change	*F* test	*R* ^2^ change	*F* test	*R* ^2^ change	*F* test
Age	0.001	0.342	0.002	0.382	0.006	1.088	0.000	0.092
Gender	0.001	0.158	0.001	0.160	0.001	0.225	0.000	0.091
Identifier	0.010	0.593	0.004	0.775	0.011	0.477	0.015	0.703
Financial problem suspected	0.281	10.936***	0.044	8.114***	0.165	4.915**	0.145	4.498**
Living circumstances	0.007	0.313	0.002	0.400	0.018	0.642	0.010	0.388
Physical capacity	0.009	2.127	0.014	2.594	0.016	2.803	0.016	2.932
Mental capacity	0.294	68.621***	0.294	54.016***	0.354	63.257***	0.392	73.258***

** *P* < 0.01, *** *P* < 0.001

### Which case features explain the greatest variance in likelihood of taking action in cases of suspected elder financial abuse?

Regression analysis to predict average likelihood of taking action per vignette revealed a significant impact of the older person’s mental capacity, and two of the categories of financial problem suspected; ‘Rogue traders’, and ‘Misuse of power of attorney authority’ (Table 5). Incremental *F* tests revealed that the older person’s mental capacity, and the nature of the financial problem suspected explained a significant amount of the variance in likelihood of action judgements. However, the *R* ^2^ change value shows that the influence of mental capacity on likelihood of action is more than double that of the nature of the financial problem suspected (Table 3).

**Table 5:** Regression model to identify the factors predicting social care and health professionals’ likelihood of action elder financial abuse

Factor (Reference category)	Category	*B*	*SE B*	*P*
	Constant	46.27	8.62	0.00
Age		−0.06	0.09	0.52
Gender		0.71	1.82	0.70
Identifier of abuse (you)	Family	−2.59	2.59	0.33
	Professional	−0.22	2.34	0.93
	Subject	−2.93	2.52	0.26
	Friend	−2.66	2.28	0.26
Financial problem suspected (A relative concerned about loss of inheritance)	Change to the person’s will	5.18	3.14	0.11
	Stealing	2.90	3.01	0.34
	Anomalies in accounts or bills	3.84	3.07	0.22
	Rogue traders	11.35	3.46	0.00***
	Misuse of POA authority	10.24	3.13	0.00**
	Anomalies between finances and living conditions	−1.90	3.04	0.54
Physical capacity		1.91	1.08	0.09
Mental capacity		8.84	1.03	0.00***
Living circs (own home)	Care package	3.05	2.71	0.27
	With family	−0.34	2.63	0.90
	Sheltered	1.52	2.42	0.54
	Residential care	2.57	3.24	0.44
	Nursing home	2.85	3.62	0.44

*R* ^*2*^ = 0.89 (*P* < 0.001)

*POA* power of attorney

***P < 0.001; ** *P* < 0.01; * *P* < 0.05

#### Comparing the influence of factors on social care and health professionals’ likelihood of action judgements

Following the same process as for regression at the overall level, regression analysis was also conducted to identify any distinctions between factor weighting by professional group. The results for regression analysis for the two groups separately with likelihood of action as the predicted variable were very similar to the overall regression findings, and the factors identified as significant by incremental *F* test analysis also followed the same pattern as the overall results (Table 4).

### What is the relationship between certainty that abuse is occurring, likelihood of taking action and action taken?

#### Certainty of abuse, likelihood of action and number of actions taken

There was a strong positive correlation between average certainty and likelihood of taking action (*r* = 0.98, *n* = 50, *P* < 0.001), suggesting a higher certainty of abuse was accompanied by a higher likelihood of action being taken. However, a paired sample *t* test showed that there was a significant difference between average certainty of abuse (M = 54.80, SD = 14.14) and likelihood of action (M = 63.32, SD = 17.16), (*t*(49) = −13.38, *P* < 0.001) indicating that the likelihood of participants taking action was generally higher than the level of certainty of financial abuse.

Social care and health care professionals’ ratings for certainty were very similar (means = 55.22 and 54.80 respectively, *t*(49) = 0.56, ns). However, social care participants’ ratings for likelihood of action (M = 66.50) were significantly higher than health care participant ratings (M = 60.21) (*t*[49] = 7.09, *P* < 0.001). Thus, social care professionals judged that they would be more likely to act than did health care professionals, even though their certainties of abuse were similar.

There was a strong positive relationship between certainty of abuse (*r* = 0.96, *n* = 50, *P* < 0.001), likelihood of taking action (*r* = 0.99, *n* = 50, *P* < 0.001) and the number of actions selected.

#### Professional group and number of actions taken

The average (mode) number of actions selected for each scenario over all participants was 3 out of a maximum possible of 6. A can be seen in Table 6, social care professionals indicated that they would take a greater number of actions in response to each scenario than health professionals (modal scores of 3 versus 2 actions). Social care professionals chose ‘Call a strategy/team meeting’, ‘Consult with outside organizations’ and ‘Implement safeguarding procedures’ significantly more often than the health care professionals.

**Table 6:** Independent sample *t* test results

Group	Monitor	Gather information	Consult internally	Strategy meeting	Consult outside	Implement safeguarding
Social care (*n* = 70) (%)	54.12	74.66	52.68	28.8	38.96	24.68
Health (*n* = 82) (%)	52.46	67.42	45.54	18.78	28.58	18.10
*t* test	*t*(96) = 1.12	*t*(98) = 1.60	*t*(98) = 1.75	*t*(98) = 3.51	*t*(98) = 2.93	*t*(89) = 2.35
	*P* = 0.27	*P* = 0.11	*P* = 0.08	*P* < 0.001	*P* < 0.01	*P* < 0.05
	*r* = 0.11	*r* = 0.16	*r* = 0.17	*r* = 0.33	*r* = 0.28	*r* = 0.24

The percentage of times each action was selected on average by social care and health professionals

Vignette *n* = 50

## Discussion

The project as a whole explored the utility of the ‘bystander intervention’ model to explain why elder financial abuse often goes unreported. This study aimed to identify the factors that have the greatest influence on UK social care and health sector professionals’ certainty that an older person is being financially abused, their likelihood of intervention, and the type of action most likely to be taken.

### Certainty and likelihood of action

For both social care and health sector professionals, only two factors had a significant influence on certainty of abuse and likelihood of taking action: these were the nature of the financial problem and mental capacity. Vignettes involving rogue traders and misuse of power of attorney, compared to anomalies between finances and living conditions and anomalies in accounts or bills, were associated with higher certainty that abuse was occurring and a greater likelihood of taking action. Our findings that only two factors accounted for most of the variance in decision-making is in line with other research showing that only a few key features of cases influence judgments (Kahneman and Frederick 2005).

The finding that social care and health professionals are much more likely to decide that financial abuse is definitely taking place, and even more likely to act if the victim is mentally incapacitated, is both readily explicable and could be seen as a matter for serious concern. The greater emphasis on mental capacity in terms of the judgement to act may be to do with the need to safeguard an individual perceived to be more vulnerable. Cases where an older person has poor mental capacity could be perceived to reflect an emergency, and therefore, there is a greater need for more decisive action. It could also be that defining abuse is more difficult if the older person has full mental capacity, and is aware and perhaps even potentially complicit in the financial abuse, for example in cases where the abuser is a loved relative who is only seen at times of financial exploitation or even outright theft.

Determining the most urgent cases, based on mental capacity could, of course, be reflective of the pressure on professionals to direct services where they are needed most. This issue was raised in Phase I of data collection when professionals explained the impact of resource and time limitations as a factor that could make taking action in cases of suspected elder financial abuse very difficult (Davies et al. 2011; Gilhooly et al. 2013). Another possible implication of this focus on reduced mental capacity is that professionals are less likely to take action in cases where the victim has full mental capacity and may not consent to intervention, either because of embarrassment or because the perpetrator of the financial abuse is known to the victim.

Although declining mental capacity has been suggested as a risk factor for elder abuse generally, (Kemp and Mosqueda 2005), it could be regarded as somewhat worrying that (lack of) mental capacity had such a strong influence on both certainty of abuse and likelihood of taking action. After all, we might want financial abuse to be detected and prevented well before people become mentally incapacitated. However, it must be kept in mind that in the vignettes there were three distinct levels of mental capacity (fully mentally aware, at times slightly confused, and extremely confused and forgetful) so it is perhaps not surprising that mental capacity came out so clearly in relation to certainty of abuse and likelihood of taking action. In real life professionals are faced with cases which are much more complex and in which incapacity might be episodic and might vary considerably from day to day or in relation to medications. In real life, therefore, it might be that mental capacity, precisely because it is less clear-cut, is not such a strong determinant of decision-making.

The finding that there were two categories of financial problems where certainty of abuse and likelihood of action was rated significantly higher—rogue traders and misuse of power of attorney—has implications for training. Firstly, professionals should remain particularly alert to these two sorts of financial problems and the potential they suggest for abuse. More importantly, training must also address the fact that financial abuse can be more subtle and professionals need to be made aware of, and have training in, how to address these more subtle forms of financial abuse, particularly financial abuse perpetrated by family members, friends and neighbours.

Misuse of power of attorney and what is known as rogue trading raise interesting issues about the definition of elder financial abuse. It is sometimes argued that these are both crimes and, hence, there is no need for the term elder financial abuse. It may be that social care and health sector professionals weigh these types of financial abuse more heavily in decision-making precisely because they represent crimes rather than the more subtle forms of financial abuse that are perpetuated by relatives and other people known and trusted by the victim of the financial abuse.

### Certainty, likelihood of action, and actions taken

Although the bystander intervention model would lead to an expectation of a significant correlation between certainty that financial abuse was taking place and likelihood of action, what somewhat surprised us was that likelihood of taking action was greater than certainty that financial abuse was taking place. However, the finding that participants from social care were more likely to take action, and to take stronger actions, could be because in the UK those in social care are tasked with and trained for adult safeguarding. In terms of the bystander intervention model, we surmise that social care professionals are more likely than non-social care professionals to decide (stage 3 of model) that the situation is a personal responsibility and (stage 4 of the bystander model) have the knowledge, and possibly some training, to deal with the it.

### Implications for theory development

Decision-making in relation to the detection and prevention of elder financial abuse was explored in this project through the lens of the bystander intervention model. Prevalence studies suggest that financial abuse may be the second most common type of elder abuse, though definitional problems make it difficult to accurately estimate prevalence (De Donder et al. 2011a, b). Moreover, it is suggested that reported financial abuse is only the ‘tip of the iceberg’ indicating that for some reason, financial abuse is often detected but no action is taken. At any one of the five stages of our professional bystander intervention model decisions could be taken that prevent abuse coming to the attention of those in a position to intervene. Exploring how professionals’ decision-making via the bystander intervention model has been, we believe, instructive in a number of ways.

Firstly, the bystander intervention model suggests that people will be less likely to take action in cases of suspected elder financial abuse where there is uncertainty that abuse is occurring. In Phase II we found that certainty of abuse and likelihood of taking action were highly correlated. However, we also found that likelihood of taking action was greater than certainty that abuse was taking place. Thus, it appears that many participants think it best to play safe and take action even if uncertain.

The third step in our proposed bystander intervention model is assuming responsibility for acting when financial abuse seems certain. In this study we also found that participants from the social care sector were more likely to indicate that they would take action compared to those from health care and that health care participants chose what we called ‘less strong’ actions. These sector differences are interesting and readily explicable given that in the UK adult safeguarding is primarily the responsibility of the social services. Moreover, in Phase I of the project we found that general practitioners often detected financial abuse, but that doctor-patient confidentiality prevented them reporting the case to adult safeguarding teams or even reporting to the police (Davies et al. 2011; Gilhooly et al. 2013). In Phase I of the project several cases were described in which patients were reported as asking their doctors not to reveal the abuse because the perpetrator was a family member on whom the older person was dependent.

The fourth stage of the bystander model is deciding that one has the skills to act. Again Phase I was instructive in that participants revealed that sometimes they did not know what to do when confronted with a case of financial abuse (Davies et al. 2011; Gilhooly et al. 2013). In countries with mandatory reporting of abuse suspicions it is presumably obvious what people must do. The UK, however, does not hold any member of the public or any professional responsible for reporting suspected abuse.

Finally, the fifth stage of the professional bystander intervention model is taking some action. There could be many barriers to taking action. Phase II did not directly address the issue of barriers to action, but it has provided interesting information about which factors do and do not play a key role in the likelihood that a social or health sector worker will intervene in some way when abuse is suspected.

More research is, of course, needed. Only a small number of factors were varied in our study and there were only three dependent variables; moreover, in many real-life cases there may be considerably less information on which to base a decision. We also need to investigate the relationship between what social care and health sector professionals say they could do when confronted with a case raising suspicions of elder financial abuse, and what they actually do. Decision-making by other professional groups also needs to be investigated, for example, the police. However, we are of the view that the bystander intervention model has considerable potential for studying the decision-making of both professionals and the public in relation to not only elder abuse, but even neglect in hospitals and care homes.

The present study indicates that, as in other decision making areas (Dhami and Harries 2001), only a small number of factors enter into decisions regarding financial abuse. Questions about the underlying cognitive processes that use the information in the factors are not addressed here. It could be, for instance, that participants do actually weight and combine factors in a linear and compensatory fashion, as proposed by social judgment theories (Cooksey 1996) or simple non-compensatory rules or heuristics (Gigerenzer and Todd 1999; Smith and Gilhooly 2006) may better describe cognitive processing in this area. Further studies, using methods tailored to reveal underlying processes, such as think-aloud procedures (Ericsson and Simon 1993; Gilhooly and Green 1996) will be required to address such cognitive level questions.

## Study limitations

Recruitment difficulties may have resulted in a sample biased towards professionals with a specific awareness and interest in elder financial abuse. The recruitment of health care professionals in particular was challenging, as a low response rate was achieved from approaches from the PCRN to general (medical) practices about the research. Although recruitment continued until the required sample size had been reached, this resulted in a high proportion of occupational therapists as part of the health professional sample. The difficulties encountered could reflect the, perhaps low, relevance of the research topic to the health sector, despite the growing emphasis of awareness of elder abuse. Perhaps the incentive for participation was insufficient.

Participants in this study had to log onto the web to participate. The study was conducted on-line because we were informed that this would be less disruptive to participant’s normal work and could be done by participants at home; doing an on-line survey increased our chances of obtaining approval from employers. However, this too may have biased the sample. Finally, we excluded from the analysis those participants who initially began the task but did not complete. It was necessary for the analysis to have a minimum of 50 complete vignettes. The vignette survey was fully automated and deleted those participants who did complete the task. This meant that we had no information on the characteristics of those who did not wish to participate or on those who started but did not finish.

Future research may need to allow for an extended recruitment period and/or greater participant incentives to create a more balanced representation of job roles within the sample. It may also be of interest to directly ask professionals if they feel it is their responsibility to take action where abuse is suspected as well as certain. We did not ask directly about perception of personal responsibility for two reasons, to limit the number of dependent variables to three and because piloting at indicated that participants from these two professional groups would almost always say that they would accept responsibility for acting where financial abuse was certain. In hindsight, however, leaving out a question of this nature was problematic given our interest in viewing the detection and prevention of elder financial abuse through the lens of the bystander intervention model.

## Concluding comments

Although mental incapacity might be a risk factor for financial elder abuse, the finding that mental capacity was a key determinant of judgements that both certainty that abuse was taking place and likelihood of intervention by health and social care professionals is concerning; prevention of financial elder abuse requires that such abuse is detected well before the older person loses mental capacity. It was, however, reassuring to find that, although highly correlated, likelihood of acting was greater than certainty of abuse, indicating that uncertainty does not prevent social care and health sector professionals from acting to stop abuse. Nevertheless, the finding that health sector workers were less likely to act when certain that financial abuse was taking place needs perhaps to be addressed. It may be that mandatory reporting is needed in the UK to help health sector workers overcome the many barriers to reporting abuse that they uncover as part of their work. Training to detect elder abuse could help. Using the bystander intervention model to explore why it is that professionals often make decisions that delay or even prevent financial abuse from coming to the attention of those in a position to act could be a useful tool in training.
